# Design and Definition of a New Decision Support System Aimed to the Hierarchization of Patients Candidate to Be Admitted to Intensive Care Units

**DOI:** 10.3390/healthcare10030587

**Published:** 2022-03-21

**Authors:** Manuel Casal-Guisande, Alberto Comesaña-Campos, Jorge Cerqueiro-Pequeño, José-Benito Bouza-Rodríguez

**Affiliations:** Department of Design in Engineering, University of Vigo, 36208 Vigo, Spain; jcerquei@uvigo.es (J.C.-P.); jbouza@uvigo.es (J.-B.B.-R.)

**Keywords:** catastrophe medicine, intensive care unit, medical decision-making, triage, hierarchization, vague fuzzy set

## Abstract

The triage processes prior to the assignation of healthcare resources in hospitals are some of the decision-making processes that more severely affect patients. This effect gets even worse in health emergency situations and intensive care units (ICUs). Aiming to facilitate the decision-making process, in this work the use of vague fuzzy numbers is proposed, aiming to define a multi-attribute patient hierarchization method to be used in emergency situations at hospital ICUs. The incorporation of fuzzy models allows for modelling the vagueness and uncertainty associated with decision criteria evaluation, with which more efficient support is provided to the decision-making process. After defining the methodology, the effectiveness of this new system for patient hierarchization is shown in a case study. As a consequence of that, it is proved that the integration of decision-support systems into healthcare environments results to be efficient and productive, suggesting that if a part of the decision process is supported by these systems, then the errors associated with wrong interpretations and/or diagnoses might be reduced.

## 1. Introduction

The assignation of healthcare resources in clinical environments is in itself a key decision that directly affects the patients involved [1,2,3]. The medical team must assess several criteria when performing triage work in order to prioritize the assistance to certain patients according to an objective interpretation of their general physical status. This situation gets even worse in health emergency situations, such as the one which the world population has been suffering since 2019 when the first SARS-CoV-2 disease cases were reported in Wuhan (China). During the still-ongoing pandemic, which since that year has spread quickly to other countries [4], there was a general increase in the pressure on hospital services that forced them to prioritize the patients in front of the scarcely available resources. Therefore, and in spite of the efforts made by countries, the health resources and the care units were not enough to attend to the sudden demand, causing many patients not to be given appropriate care, and even leading some of them to a premature death. According to Rubio et al. [3], in situations involving a sudden increase in the demand for intensive care combined with severe difficulties in incorporating new care resources, ‘catastrophe medicine’ scenarios might appear in which difficult decisions must be made related to the assignation of resources and the prioritization of patients. Thus, in critical or sanitary collapse situations, decisions such as the removal of mechanical ventilation, usually made when this procedure is no longer therapeutically indicated or when it is specifically requested by the patient himself or their relatives [1], must be made according to all the fundamental guarantees and rights by resorting to new triage procedures. These new procedures will require considering a series of medical factors that will eventually determine the priority for the treatment. Several intervention guidelines and protocols have appeared during the past year 2020 [1,2] relative to the application of those factors in emergency situations, in which a recommendation is explicitly made towards the use of decision support systems.

In this work, the elemental proposal made by the authors in Casal-Guisande et al. [5], which addressed the concept design of a decision support tool that would allow for the determination of the priority of candidates to be admitted to Intensive Care Units in catastrophe medicine situations, is presented and developed in detail. By making use of vague fuzzy numbers, this methodology aims to combine the opinion of several professionals, taking into account multiple criteria and providing a care hierarchy for the patients based on these criteria.

### 1.1. General Triage Guidelines and Protocols for Sanitary Collapse Situations

During the year 2020, several countries worldwide issued new guidelines aiming to establish a set of protocols [1,6] about triage procedures to be applied in hospital emergency services in order to help the medical professionals to make decisions related to the prioritization of care given to the incoming patients. This process involves enormous responsibility combined with undeniable ethical and legal implications [1].

In general, said guidelines take into account a series of aspects that are summarized in Table 1 [1,2] which are developed in more depth in the work by Joebges and Biller-Andorno [1].

The patients are generally stratified according to four groups of admittance priority to Intensive Care Units (ICU) depending on their characteristics, which are listed in Table 2. During a health crisis situation, only the admittance to ICU services of patients having the 1st or 2nd priority level will be considered, while 3rd and 4th priority-level patients will not be considered for admittance [3]. In situations where resources are still more limited, then 1st priority-level patients prevail over 2nd-level ones.

### 1.2. Hierarchization Processes

Generally speaking, the decision-making process involves the determination of the correct answer to a certain problem, which might deal with a choice or an optimization [7,8], for example. To do so, the decider must consider several different decision criteria that are intimately linked to the problem. Multi-criteria decision methods [9] are thus framed, which are usually classified either as multi-objective (when searching for a potential optimal solution) and multi-attribute (when looking for the best decision among a limited number of alternatives). Multi-attribute methods are widely used in medical environments, providing trusted environments where decisions can be made once the criteria have been established. Thus, the analytical hierarchical process (AHP) [10,11,12,13,14,15,16], TOPSIS [17,18,19], MAUT [10,16,20,21], PROMETHEE [22,23,24,25] and VIKOR [26,27,28] methods, among others, are applied to decision management in medical–sanitary environments. However, the increasing need for incorporating and controlling inaccuracy in information and the judgments in decision making have popularized the use of methods that are capable of controlling such decision uncertainty. The developments and advances in artificial intelligence algorithms have allowed the reasoning and learning models to give support to decision support systems [29], thus allowing them to diversify and complement their usage. Starting from—either symbolic or statistical—inferential processes, approaches such as machine learning, deep learning or expert systems have the inherent capability to find probabilistic and/or logical relationships that allow for limiting the uncertainty and reducing the risk associated with decision making [29,30,31,32,33,34,35]. However, the use of these techniques requires advanced learning models, together with complex representations of reasoning or learning that implies the need for the availability of a large starting dataset, or for experts in charge of processing such data and elaborate association rules. That is the reason why this work will make use of more direct and simple approaches that do not depend on data or on experts while, however, they might offer an easy management of uncertainty. There are already many works describing a probabilistic management of uncertainty regarding its random and epistemic definition which might be applied to decision-making processes [7,36]. Nevertheless, their use is conditioned to environments where information is perfectly structured, complete and truthful. In uncertain environments, with information being biased, only partially truthful and contradictory, probabilistic models find larger difficulties in reaching valuable accuracy levels [37]. Thus, in addition to the capabilities of probability-based methods, the fuzzy-logic methods [38,39] allow for the control of uncertainty and inconsistency in decision judgments, controlling at the same time the quantification of the weighting of criteria at the time of choosing the best decision. Extending the classical fuzzy set concept, vague fuzzy numbers [40] are grounded on the definition of an interval where the membership function would be found that, in turn, determines the degree of membership of a certain value to the fuzzy set with which it is associated. This interval is characterized by a truth-membership function ($t_{A}$) and a false-membership function ($f_{A}$), which set the limits between, respectively, the affirmation and the negation of the membership.

Vague numbers extended uncertainty control within fuzzy logic, and they are similar to other approaches based on this logic as might be the intuitionistic numbers developed by Atanassov [41,42], characterized by defining a membership function ($\mu_{A}$) and a non-membership function ($v_{A}$) for each fuzzy set.

Table 3 shows the analogy that exists between the intuitionistic and the vague sets.

By combining the vague numbers algebra with aggregation and hierarchization operators, Xu and Yager [43] enunciated a series of hierarchization operators, based on vague or intuitionistic numbers, that have extended the multi-attribute hierarchization concept. Said operators are summarized in Table 4, with $\tilde{a}=\left[t_{\tilde{a}},1-f_{\tilde{a}}\right]$ being a vague fuzzy number and $\omega ={\left(\omega_{1},\omega_{2},\ldots ,\omega_{n}\right)}^{T}$ being a weighting vector associated to the elements to be hierarchized.

These operators allow the aggregation of the different vague number matrices to be carried out. It is also essential, however, to introduce the score concept, which allows the defuzzification process to be performed, the expression of which is shown in Equation (1).


(1) $$S\left(\tilde{a}\right)=t_{\tilde{a}}-f_{\tilde{a}}$$

## 2. Materials and Methods

### 2.1. Conceptual Design of the System

**Figure 1:**
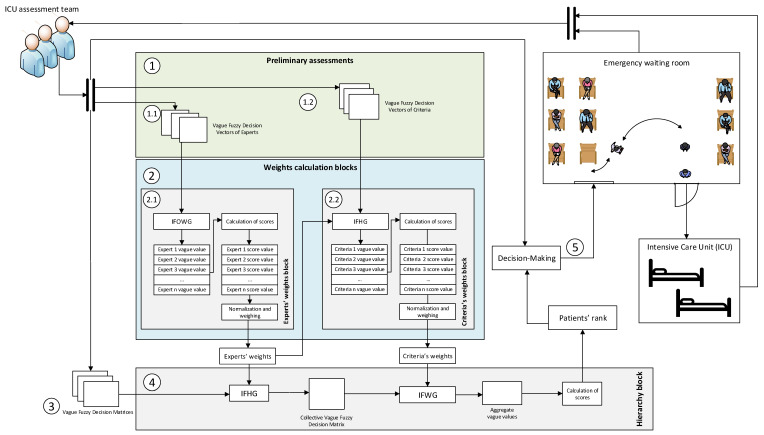
Flow diagram for the decision support system. This diagram shows the different flows of information across the system stages presented in Table 5. Label 1 is related with the preliminary assessments, label 2 with the weightings calculation block, label 3 with the patients’ status assessment, label 4 with the hierarchy calculation block, and finally, label 5 is related with decision-making.

The starting point will be the proposal initially presented by the authors in their 2020 conference paper [5]. Figure 1 shows in detail the flow diagram of the proposed system.

Next, in Table 5, a brief description of the different stages in Figure 1 is provided.

### 2.2. Implementation of the System

The system described in Section 2.1 considers the different stages that make possible the compilation and processing of the different assessments produced by the sanitary experts from the ICU admittance team, which will be expressed using vague fuzzy numbers [10] because of their proven capabilities to manage vagueness and to represent uncertain information, as well as to control the uncertainty associated to the process itself for the determination of the membership functions. From them, it will be possible to determine the priority of patients by using a series of operators derived from the vague fuzzy set concept as described in Section 1.2.

#### 2.2.1. Weightings Calculation Blocks

Starting from the preliminary assessments established by the experts, expressed using vague fuzzy numbers, it is intended, on the one hand, to determine the worth and importance of each one of the admittance team members, and on the other hand, to measure the importance of each of the criteria used to assess the patients’ admittance, as seen next.

- Experts’ weightings calculation: Taking the values of the Vague Fuzzy Decision Vectors of Experts, first the IFOWG operator [43] is applied to determine the weighting vector of the experts $\omega_{j}$ by means of the method based on the normal distribution [44]. As it is not possible to objectively weight the experts’ assessments, the normal distribution is taken as a common representation of natural scoring processes. By means of IFOWG it is possible to aggregate the opinion of the different experts on each one of them. Later, the score [43] associated to each expert is calculated, which will be normalized and weighted in the [0–1] range, so that the sum of all of them is equal to 1.
- Criteria’s weightings calculation: The block associated to the calculation of the criteria weightings is similar to the experts’ one. The only difference lies in the operator being used, in this case the IFHG [43] operator that makes use of the previously calculated vector $\;\omega_{j}$ of the experts’ weightings. The use of this hybrid operator allows for the combination of the effects of the direct weightings, making use of the experts’ weighting vector, and organized by considering a normal distribution associated to the assessment of the criteria by these same experts. Same as in the previous case, the score is later calculated, and after that the normalization and weighting of the different weights obtained is carried out.

#### 2.2.2. Hierarchy Block

At the same time, the experts will assess the different patients’ status, and each expert’s Vague Fuzzy Decision Matrix is to be filled-in for every patient. As shown in Figure 1, these matrices are first processed by means of the IFGH operator [43], taking into account the expert weightings as for $\omega_{j}$. The Collective Vague Fuzzy Matrix, which represents the joint assessment by the different experts of each one of the patients’ statuses, is obtained as an output of said operator.

After that, the IFWG operator is applied to the Collective Vague Fuzzy Matrix, taking into account the expert weightings as for $\omega_{j}$. The output of said operator produces the Aggregate Vague Values.

The calculation each patient’s score is performed later. The patients are then ordered based on their obtained score, thus establishing a priority ranking among them.

## 3. Simulations and Results

With the goal of showing the operation of the system, in this section a case study is presented which takes into account five experts (E_1_, E_2_, E_3_, E_4_ and E_5_), five criteria (C_1_, C_2_, C_3_, C_4_ and C_5_) and five patients (P_1_, P_2_, P_3_, P_4_ and P_5_).

The starting data for the case study will be synthesized and adapted from different information sources and datasets, and therefore it will not be obtained from real application environments. The main goal of this case study is to demonstrate the internal operation of the decision support system, representing its different stages and showing the data-collection process and the structure of such data. As such, it was considered that the source of data would not be a determining factor in the system’s performance and outcomes because, actually, it heavily depends on the users and their valuations. Thus it is suggested that, when facing similar circumstances, the environment experts might produce different, and even contradictory, information. The scope of the system will precisely be to be used as a basis to model information and as a vehicle to reach the solution, a topic that will be described next.

### 3.1. Preliminary Assessments

Firstly, the experts from the sanitary team will assess each other using vague fuzzy numbers, thus producing the Vague Fuzzy Decision Vectors of Experts, which are shown grouped together in Table 6. The values on each table row are associated to the assessment made by each expert on their team mates.

After that, the experts will also assess the importance of the criteria to be employed, also making use of vague fuzzy numbers, thus producing the Vague Fuzzy Decision Vectors of Criteria, which are shown grouped together in Table 7. The values on each table row are associated to the assessment made by each expert on the different criteria.

### 3.2. Weightings Calculation Blocks

After performing the assessments explained above, it is time to proceed to the calculation of both the experts’ and the criteria’s weightings.

#### 3.2.1. Determination of the Experts’ Weightings Vector

In order to proceed to the calculation of the experts’ weightings, the starting data will be that shown in Table 6 below, obtaining first the vague fuzzy vector of the aggregated opinions for each expert, which can be seen in Table 8, after applying the IFOWG operator.

After that, the score is calculated for each expert producing the values shown in Table 9 which, after being conveniently normalized, determine the experts’ weightings as shown in Table 10 below. The expert with number 4 results to be the most important, followed by those with numbers 5, 2, 3, and lastly the expert with number 1.

#### 3.2.2. Determination of the Criteria Weighting Vector

In order to calculate the weightings of the different criteria, the starting data will be the assessments made by the experts on the different criteria, shown in Table 7, to which the IFGH operator will be applied. In the Table 11 it is possible to see the aggregated vague values vector for each criterion.

After the criteria values have been aggregated, the scores (Table 12) are calculated and these values are later normalized to determine the criteria weighting vector, shown in Table 13.

### 3.3. Patients’ Status Assessment

Once the experts and the criteria weightings have been determined, it is then possible to carry out the intensive work needed. Table 14, Table 15, Table 16, Table 17 and Table 18 show the assessments made on each patient’s status by the different experts, taking into account the established criteria and using vague fuzzy numbers.

### 3.4. Hierarchy Block

**Figure 2:**
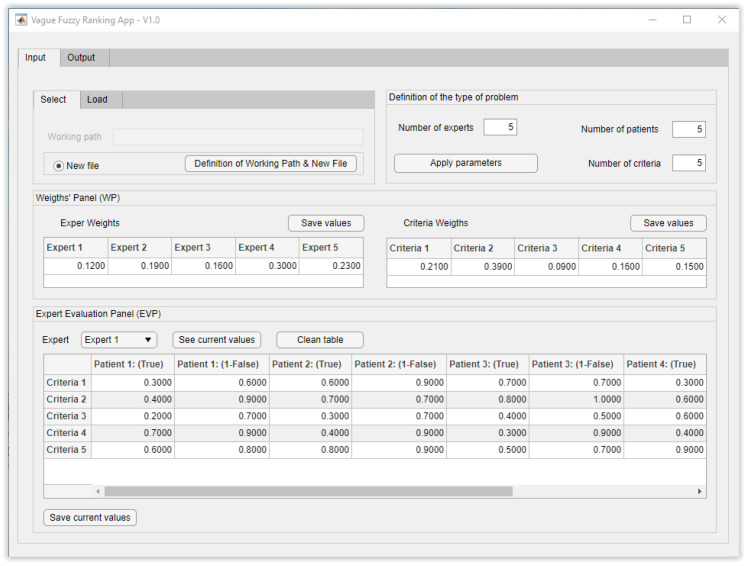
‘Definition of the type of problem’ screen with data already loaded.

After the experts have assessed the different patients’ statuses, it is possible to proceed to their hierarchization, that is, to determine the priority order for admittance to the Intensive Care Unit. In order to ease that process, an application has been developed on MATLAB© R2021a that incorporates a fully functional interface built on the App Designer software, which allows the calculations that the hierarchization of the patients involves to be performed fast and easily. Figure 2 shows how the problem data have been loaded on the ‘Definition of the type of problem’ screen, introducing the experts and the criteria weightings into the ‘Weight’s Panel’, as well as the expert assessments into the ‘Expert Evaluation Panel’. 

Starting from this information, and according to the description made in Section 2.2, the hierarchization of the patients is carried out, thus obtaining the Collective Vague Fuzzy Decision Matrix, that is, the matrix conjointly representing the assessments made by the different experts. Such matrix is shown in Table 19.

The IFWG operator is applied next, which actuates on the Collective Vague Fuzzy Decision Matrix and allows for the determination of the Aggregate Vague Values that are shown in Table 20.

**Figure 3:**
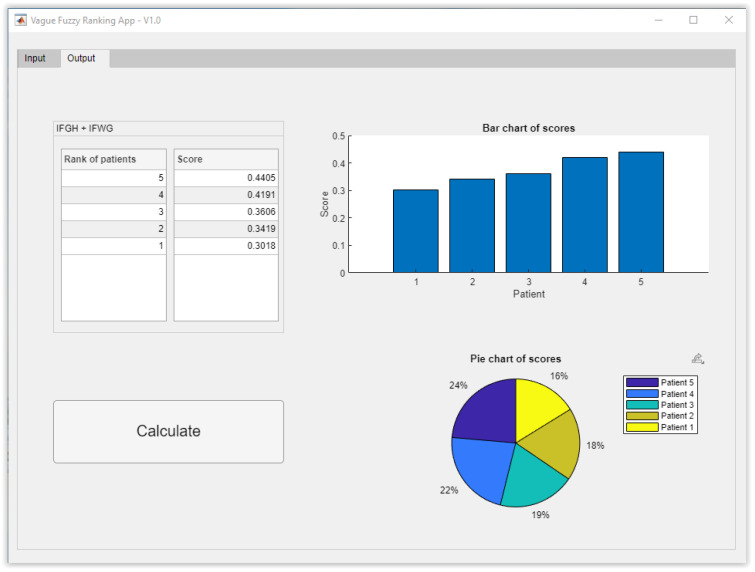
Hierarchy calculation module.

Finally, the score calculation is performed, from which it is possible to determine the patients’ hierarchy. Figure 3 shows the application’s calculation panel in which the hierarchization results can be seen, ranking the patients according to decreasing score values, thus observing that the patient with the highest preference is P_5_, followed, respectively, by P_4_, P_3_, P_2_ and P_1_.

Once the hierarchy has been determined, the team of experts receives the recommendation and must then make a decision, taking into account such recommendation provided by the system.

## 4. Discussion

The use of hierarchization and prioritization models associated to decision support systems is common in sanitary environments. By using multi-attribute approaches, combining the assessment of multiple criteria, they allow objective classifications of the decision alternatives to be established. In this work a multi-attribute approach has been applied to the care prioritization of patients at hospital ICUs. By incorporating the existing triage models and involving the sanitary experts in the processes, a system has been defined that allows the experts’ assessments to be collected by means of vague fuzzy numbers and for them to be combined by means of aggregators based in weighting models that are specific to those criteria and experts. The use of vague numbers allows both the random and the epistemic uncertainty to be limited [7,45] within the assessment process, as these assessments get their usual determination pattern complemented with an indeterminacy one. The successive assessments of the criteria need to answer both on the affirmation and on the negation of the case to be evaluated under the consideration of a certain criterion, extending the control on the uncertainty associated to that answer. In the same way, the aggregation of these assessments produces new vague numbers whose final defuzzification will successively determine the experts, criteria and patients’ hierarchization weighting vectors. When compared to the classical multi-attribute methods, the management of uncertainty is already one of the differentiating aspects. Compared to other methods that allow the implicit management of uncertainty, it is the use of fuzzy vague numbers in itself which constitutes a novel contribution. By generalizing the usefulness of fuzzy logic with vague numbers, its capability to represent the vagueness and imperfection of language are generalized too, a key factor with interpretative decisions such as those associated to all triage processes.

The choice for the different operators arises from a need for practicality and objectivity. The use of ordered models in the determination of the experts’ weightings is justified because it is not possible to hierarchize them in an unquestionable way, so it is intended that their crossed opinions resemble a normal distribution, that is, the effect of unbalanced and asymmetric opinions will be minimized by reducing the effect or the assessments in the ends of the assessment interval. In the case of the criteria weightings, it will be possible to make use of the previously calculated experts’ weightings, so a hybrid operator will be used that will combine the ordered effects associated with the criteria assessment with direct weightings related to the importance of the experts. The final determination of the hierarchization of the patients will be developed in the same way, making a direct use of the previously calculated weighting vectors.

In spite of the previously described considerations and the correct operation that is expected from the presented decision support system, there are some issues that limit its applicability, usage potential and reliability. Certainly, fuzzy logic-based multi-criteria methods might easily produce a hierarchization of variables by considering several criteria. However, that requires establishing a series of subjective assessments represented by a vague fuzzy number that is associated to each variable, in this work being the experts, criteria and, finally, the patients. This demands performing an abstraction that indicates the membership and non-membership degrees of the variable to a fuzzy set which, even if it implicitly delimits uncertainty, explicitly it makes necessary an effort to properly represent the vagueness of the information by aiming to formalize and diversify the systems’ information. The expert in charge of inputting the initial data must avoid contradictions and biases at all costs, and of course not to lie in a deliberate or tricky way. Without these conditions, the control of uncertainty, even with the use of fuzzy logic approaches, would result to be inefficient and shorthanded. On the other hand, the use of language symbols, qualifiers and quantifiers, associated to the different membership functions, imply an unavoidable increase in the subjectivity of the decision-making process. The reasons for using some specific functions instead of others, or some common-language qualifier instead of others, will have direct and immediate effects on the proposed hierarchization, and therefore on the decision made itself. There will unavoidably exist a dependence of the system on its users, a matter that is not minor, and therefore it is not possible as of yet to extrapolate its usefulness to other different use environments.

## 5. Conclusions

In this work, a novel decision support system has been presented that allows to speed up the triage processes associated to the hierarchization of patients at hospital ICUs, especially when considering sanitary emergency situations. The use of multi-attribute methods combined with assessments based on fuzzy vague numbers has allowed uncertainty to be incorporated into the system, as well as the representation of information and the interpretation of the decision criteria by the sanitary experts to be facilitated. Even if the results derived from the case study seem to be promising, this system must be evolved to incorporate metrics for accuracy and error, so that its recommendations might properly be compared with actual objective results. The expected incorporation in the future of decision support intelligent systems will improve the overall operation of the current system and will help to overcome the limitations associated with the diversification and formalization of the necessary information.

## Figures and Tables

**Table 1:** Main criteria considered for the assessment of patients’ status in sanitary collapse situations. Elaborated from the work by Joebges and Biller-Andorno [1].

Equity.
Maximizing benefit, broadly understood as the maximization of benefit for the largest possible number of patients according to the available resources.
Considering the patient’s age and life span.
Other additional criteria.
Patient’s will.
Therapeutic ceiling, understood as the termination of therapy.
Additional recommendations: generally advocating for transparent decision-making processes. In the case of the UK, the use of decision support tools is recommended when they are available.
Periodical re-evaluation of the patient’s status.
Who decides? The recommendation is generally given that decisions be made by expert teams from the health field, with at least two professionals involved in the procedure.

**Table 2:** Priority groups for ICU admittance. Elaborated from the work by Rubio et al. [3].

1st Priority Level	2nd Priority Level	3rd Priority Level	4th Priority Level
Applicable to patients who need to be intensively monitored and who must be provided with intensive care services, such as invasive mechanical ventilation or continuous extra-renal depuration, among others.	Applicable to patients who also need to be intensively monitored, and who might demand an immediate intervention. They might require an oxygen-therapy supply, but in a non-invasive way. They might have issues in any other body organ.	Applicable to patients that have a small probability of recovery because of their base diseases. They may still be provided with palliative care.	Applicable to patients for whom ICU admission would not result in a substantial benefit because of their status.

**Table 3:** Definition of the intuitionistic fuzzy set and the vague fuzzy set.

Intuitionistic Fuzzy Set	Vague Fuzzy Set
$A_{I}=\left\{\langle x,\mu_{A}\left(x\right),v_{A}\left(x\right)\rangle |x\in X\right\}$	$A_{V}=\left\{\langle x,\left[t_{A}\left(x\right),1-f_{A}\left(x\right)\right]\rangle |x\in X\right\}$
$\mu_{A}\left(x\right)+v_{A}\left(x\right)\leq 1$	$t_{A}\left(x\right)+f_{A}\left(x\right)\leq 1$

**Table 4:** Main operators.

Intuitionistic Fuzzy Weighted Geometric (direct weighting of vague values)	$IFWG_{\omega }\left({\tilde{a}}_{1},{\tilde{a}}_{2},\ldots ,{\tilde{a}}_{n}\right)=\left[\prod_{j=1}^{n}t_{{\tilde{a}}_{j}}^{\omega_{j}},\prod_{j=1}^{n}{\left(1-f_{{\tilde{a}}_{j}}\right)}^{\omega_{j}}\right]$
Intuitionistic Fuzzy Ordered Weighted Geometric (weighting in an orderly manner of vague values)	$IFOWG_{w}\left({\tilde{a}}_{1},{\tilde{a}}_{2},\ldots ,{\tilde{a}}_{n}\right)=\left[\prod_{j=1}^{n}{\left(t_{{\tilde{a}}_{\sigma \left(j\right)}}\right)}^{\omega_{j}},\prod_{j=1}^{n}{\left(1-f_{{\tilde{a}}_{\sigma \left(j\right)}}\right)}^{\omega_{j}}\right]$
Intuitionistic Fuzzy Hybrid Geometric (combination of the two previous approaches)	$IFHG_{\omega ,w}\left({\tilde{a}}_{1},{\tilde{a}}_{2},\ldots ,{\tilde{a}}_{n}\right)=\left[\prod_{j=1}^{n}t_{{\dot{\tilde{a}}}_{\sigma \left(j\right)}}^{w_{j}},\prod_{j=1}^{n}{\left(1-f_{{\dot{\tilde{a}}}_{\sigma \left(j\right)}}\right)}^{\omega_{j}}\right]$

**Table 5:** Stages.

**Stage 1—Preliminary Assessments**
Each member of the team in charge of determining the admittance to the ICU will assess the experience and importance of each other team member, these expressed by means of vague fuzzy numbers and stored into the Vague Fuzzy Decision Vectors of Experts. Later, the experts will establish the criteria to be used to assess the admittance to the ICU taking into account the applicable protocols and recommendations. After that, each one of the experts will evaluate the importance of the different criteria, which will be also expressed using vague fuzzy numbers and will be stored into the Vague Fuzzy Decision Vectors of Criteria.
**Stage 2—Weightings Calculation Blocks**
Starting from the Vague Fuzzy Decision Vectors of Experts and of Criteria, in the 2.1 and 2.2 blocks from Figure 1 the aggregated weighting vectors associated to the experts and to the criteria are calculated by applying a series of operators. The weighting vectors calculated in this stage will be later necessary to allow the aggregation of the patients’ status assessment in Stage 4.
**Stage 3—Patients’ Status Assessment**
Each member of the admittance team will assess the status of each patient taking into account the previously determined criteria. The assessment associated to the different patients’ status, produced by each expert, will be stored into a matrix named Vague Fuzzy Decision Matrix, as shown in Figure 1.
**Stage 4—Hierarchy Block**
By applying the operators indicated in Stage 2 on the matrices obtained in Stage 3, and taking into account the weighting vectors previously obtained, it is possible to establish a ranking or patients according to the previously established assessments.
**Stage 5—Decision-Making**
Starting from the information associated to the ranking obtained as a result of Stage 4, the admittance team will decide which patients will be admitted to the ICU. It is essential to point out that there will be a periodical evaluation of both the evolution and the priority of the patients currently in the ICU.

**Table 6:** Vague Fuzzy Decision Vectors of Experts.

	E_1_		E_2_		E_3_		E_4_		E_5_	
	$t_{\tilde{a}}$	$1-f_{\tilde{a}}$	$t_{\tilde{a}}$	$1-f_{\tilde{a}}$	$t_{\tilde{a}}$	$1-f_{\tilde{a}}$	$t_{\tilde{a}}$	$1-f_{\tilde{a}}$	$t_{\tilde{a}}$	$1-f_{\tilde{a}}$
E_1_	-	-	0.7	0.8	0.5	0.8	0.9	1	0.6	0.9
E_2_	0.5	0.8	-	-	0.6	0.9	0.8	1	0.8	1
E_3_	0.5	0.6	0.8	1	-	-	1	1	0.7	0.8
E_4_	0.6	0.7	0.7	0.9	0.7	1	-	-	0.8	0.9
E_5_	0.7	0.8	0.6	0.8	0.6	0.9	0.7	0.9	-	-

**Table 7:** Vague Fuzzy Decision Vectors of Criteria.

	C_1_		C_2_		C_3_		C_4_		C_5_	
	$t_{\tilde{a}}$	$1-f_{\tilde{a}}$	$t_{\tilde{a}}$	$1-f_{\tilde{a}}$	$t_{\tilde{a}}$	$1-f_{\tilde{a}}$	$t_{\tilde{a}}$	$1-f_{\tilde{a}}$	$t_{\tilde{a}}$	$1-f_{\tilde{a}}$
E_1_	0.4	0.7	0.8	1	0.2	0.5	0.5	0.9	0.7	0.9
E_2_	0.6	0.8	0.6	0.9	0.4	0.7	0.6	0.8	0.5	0.8
E_3_	0.5	0.8	0.8	0.9	0.5	0.8	0.3	0.5	0.6	1
E_4_	0.7	0.8	1	1	0.5	0.7	0.6	0.7	0.5	0.7
E_5_	0.6	0.8	0.6	0.9	0.6	0.8	0.7	0.8	0.4	0.6

**Table 8:** Aggregation of the assessments of the different experts on each other.

E_1_		E_2_		E_3_		E_4_		E_5_	
$t_{\tilde{a}}$	$1-f_{\tilde{a}}$	$t_{\tilde{a}}$	$1-f_{\tilde{a}}$	$t_{\tilde{a}}$	$1-f_{\tilde{a}}$	$t_{\tilde{a}}$	$1-f_{\tilde{a}}$	$t_{\tilde{a}}$	$1-f_{\tilde{a}}$
0.56	0.77	0.70	0.84	0.56	0.90	0.84	0.98	0.71	0.93

**Table 9:** Experts’ scores.

E_1_	E_2_	E_3_	E_4_	E_5_
0.33	0.54	0.46	0.83	0.64

**Table 10:** Experts’ weightings.

E_1_	E_2_	E_3_	E_4_	E_5_
0.12	0.19	0.16	0.30	0.23

**Table 11:** Aggregated vague values vector for each criterion.

C_1_		C_2_		C_3_		C_4_		C_5_	
$t_{\tilde{a}}$	$1-f_{\tilde{a}}$	$t_{\tilde{a}}$	$1-f_{\tilde{a}}$	$t_{\tilde{a}}$	$1-f_{\tilde{a}}$	$t_{\tilde{a}}$	$1-f_{\tilde{a}}$	$t_{\tilde{a}}$	$1-f_{\tilde{a}}$
0.58	0.80	0.71	0.56	0.73	0.50	0.77	0.76	0.94	0.45

**Table 12:** Criteria scores.

C_1_	C_2_	C_3_	C_4_	C_5_
0.37	0.70	0.16	0.28	0.27

**Table 13:** Criteria weightings.

C_1_	C_2_	C_3_	C_4_	C_5_
0.21	0.39	0.09	0.16	0.15

**Table 14:** Expert 1’s assessments on the different patients’ status.

E_1_	P_1_		P_2_		P_3_		P_4_		P_5_	
	$t_{\tilde{a}}$	$1-f_{\tilde{a}}$	$t_{\tilde{a}}$	$1-f_{\tilde{a}}$	$t_{\tilde{a}}$	$1-f_{\tilde{a}}$	$t_{\tilde{a}}$	$1-f_{\tilde{a}}$	$t_{\tilde{a}}$	$1-f_{\tilde{a}}$
C_1_	0.3	0.6	0.6	0.9	0.7	0.7	0.3	0.6	0.8	0.9
C_2_	0.4	0.9	0.7	0.7	0.8	1	0.6	0.9	0.3	0.7
C_3_	0.2	0.7	0.3	0.7	0.4	0.5	0.6	0.9	0.4	0.5
C_4_	0.7	0.9	0.4	0.9	0.3	0.9	0.4	0.8	0.7	1
C_5_	0.6	0.8	0.8	0.9	0.5	0.7	0.9	1	0.5	0.8

**Table 15:** Expert 2’s assessments on the different patients’ status.

E_2_	P_1_		P_2_		P_3_		P_4_		P_5_	
	$t_{\tilde{a}}$	$1-f_{\tilde{a}}$	$t_{\tilde{a}}$	$1-f_{\tilde{a}}$	$t_{\tilde{a}}$	$1-f_{\tilde{a}}$	$t_{\tilde{a}}$	$1-f_{\tilde{a}}$	$t_{\tilde{a}}$	$1-f_{\tilde{a}}$
C_1_	0.5	0.7	0.7	0.8	0.5	0.7	0.5	0.6	0.7	0.7
C_2_	0.6	0.9	0.6	0.9	0.7	0.8	0.7	0.9	0.5	0.6
C_3_	0.7	0.8	0.4	0.6	0.6	0.7	0.4	0.8	0.6	0.7
C_4_	0.7	0.9	0.6	0.8	0.3	0.8	0.7	0.8	0.6	1
C_5_	0.8	0.8	0.7	0.7	0.6	0.9	0.7	0.7	0.5	0.9

**Table 16:** Expert 3’s assessments on the different patients’ status.

E_3_	P_1_		P_2_		P_3_		P_4_		P_5_	
	$t_{\tilde{a}}$	$1-f_{\tilde{a}}$	$t_{\tilde{a}}$	$1-f_{\tilde{a}}$	$t_{\tilde{a}}$	$1-f_{\tilde{a}}$	$t_{\tilde{a}}$	$1-f_{\tilde{a}}$	$t_{\tilde{a}}$	$1-f_{\tilde{a}}$
C_1_	0.6	0.7	0.8	0.8	0.6	0.9	0.6	0.6	0.8	0.9
C_2_	0.5	1	0.5	0.7	0.8	1	0.6	0.9	0.6	0.8
C_3_	0.6	0.6	0.6	0.8	0.7	0.7	0.5	0.7	0.7	0.7
C_4_	0.6	0.9	0.5	1	0.4	0.9	0.9	0.9	0.5	0.8
C_5_	0.7	0.7	0.6	0.7	0.8	0.9	0.5	0.8	0.7	1

**Table 17:** Expert 4’s assessments on the different patients’ status.

E_4_	P_1_		P_2_		P_3_		P_4_		P_5_	
	$t_{\tilde{a}}$	$1-f_{\tilde{a}}$	$t_{\tilde{a}}$	$1-f_{\tilde{a}}$	$t_{\tilde{a}}$	$1-f_{\tilde{a}}$	$t_{\tilde{a}}$	$1-f_{\tilde{a}}$	$t_{\tilde{a}}$	$1-f_{\tilde{a}}$
C_1_	0.3	0.6	0.7	0.9	0.4	0.9	0.8	0.8	0.6	0.9
C_2_	0.5	0.9	0.7	0.7	0.5	0.8	0.5	0.9	0.6	0.8
C_3_	0.8	0.9	0.3	0.9	0.8	0.9	0.4	1	0.7	0.8
C_4_	0.3	0.5	0.8	0.8	0.6	0.8	0.7	0.8	0.6	1
C_5_	0.4	0.4	0.3	0.9	0.6	0.7	0.7	0.9	0.8	0.9

**Table 18:** Expert 5’s assessments on the different patients’ status.

E_5_	P_1_		P_2_		P_3_		P_4_		P_5_	
	$t_{\tilde{a}}$	$1-f_{\tilde{a}}$	$t_{\tilde{a}}$	$1-f_{\tilde{a}}$	$t_{\tilde{a}}$	$1-f_{\tilde{a}}$	$t_{\tilde{a}}$	$1-f_{\tilde{a}}$	$t_{\tilde{a}}$	$1-f_{\tilde{a}}$
C_1_	0.5	0.7	0.6	0.9	0.2	1	0.7	0.9	0.9	1
C_2_	0.8	1	0.5	0.7	0.4	0.9	0.4	1	0.7	1
C_3_	0.7	0.8	0.3	0.9	0.7	0.9	0.7	0.9	0.6	0.8
C_4_	0.6	0.7	0.7	0.9	0.4	0.9	0.6	0.9	0.5	0.7
C_5_	0.2	0.3	0.1	0.7	0.4	0.8	0.5	0.7	0.7	0.9

**Table 19:** Collective Vague Fuzzy Decision Matrix.

	P_1_		P_2_		P_3_		P_4_		P_5_	
	$t_{\tilde{a}}$	$1-f_{\tilde{a}}$	$t_{\tilde{a}}$	$1-f_{\tilde{a}}$	$t_{\tilde{a}}$	$1-f_{\tilde{a}}$	$t_{\tilde{a}}$	$1-f_{\tilde{a}}$	$t_{\tilde{a}}$	$1-f_{\tilde{a}}$
C_1_	0.444	0.679	0.685	0.852	0.402	0.848	0.611	0.709	0.767	0.871
C_2_	0.575	0.939	0.593	0.736	0.591	0.878	0.547	0.929	0.540	0.786
C_3_	0.640	0.766	0.352	0.787	0.672	0.771	0.486	0.864	0.603	0.725
C_4_	0.566	0.764	0.632	0.860	0.414	0.847	0.634	0.847	0.554	0.916
C_5_	0.489	0.537	0.390	0.773	0.551	0.804	0.633	0.796	0.665	0.890

**Table 20:** Aggregate Vague Values.

	$t_{\tilde{a}}$	$1-f_{\tilde{a}}$
P_1_	0.536	0.766
P_2_	0.553	0.789
P_3_	0.515	0.845
P_4_	0.580	0.840
P_5_	0.608	0.832

## Data Availability

Not applicable.

## References

[1] Joebges S., Biller-Andorno N. (2020). Ethics Guidelines on COVID-19 Triage-an Emerging International Consensus. Crit. Care.

[2] Jöbges S., Vinay R., Luyckx V.A., Biller-Andorno N. (2020). Recommendations on COVID-19 Triage: International Comparison and Ethical Analysis. Bioethics.

[3] Rubio O., Estella A., Cabre L., Saralegui-Reta I., Martin M.C., Zapata L., Esquerda M., Ferrer R., Castellanos A., Trenado J. (2020). Ethical Recommendations for a Difficult Decision-Making in Intensive Care Units Due to the Exceptional Situation of Crisis by the COVID-19 Pandemia: A Rapid Review & Consensus of Experts. Med. Intensiva.

[4] Velavan T.P., Meyer C.G. (2020). The COVID-19 Epidemic. Trop. Med. Int. Health.

[5] Casal-Guisande M., Cerqueiro-Pequeño J., Comesaña-Campos A., Bouza-Rodríguez J.B. Conceptual Proposal of a Hierarchization System for Patients Candidate to Intensive Care Units in Health Catastrophe Situations. Proceedings of the 8th International Conference on Technological Ecosystems for Enhancing Multiculturality.

[6] Guidelines—International Society for Priorities in Health. https://prioritiesinhealth.org/guidelines.

[7] Herrmann J.W. (2015). Engineering Decision Making and Risk Management.

[8] Turban E., Aronson J.E., Liang T.-P. (2007). Decision Support Systems and Intelligent Systems.

[9] Ching-Lai H., Kwangsun Y. (1981). Multiple Attribute Decision Making: Methods and Applications a State-of-the-Art Survey.

[10] Adunlin G., Diaby V., Xiao H. (2015). Application of Multicriteria Decision Analysis in Health Care: A Systematic Review and Bibliometric Analysis. Health Expect..

[11] Diaz-Ledezma C., Parvizi J. (2013). Surgical Approaches for Cam Femoroacetabular Impingement: The Use of Multicriteria Decision Analysis Hip. Proceedings of the Clinical Orthopaedics and Related Research.

[12] Maruthur N.M., Joy S., Dolan J., Segal J.B., Shihab H.M., Singh S. (2013). Systematic Assessment of Benefits and Risks: Study Protocol for a Multi-Criteria Decision Analysis Using the Analytic Hierarchy Process for Comparative Effectiveness Research. F1000Research.

[13] Pecchia L., Martin J.L., Ragozzino A., Vanzanella C., Scognamiglio A., Mirarchi L., Morgan S.P. (2013). User Needs Elicitation via Analytic Hierarchy Process (AHP). A Case Study on a Computed Tomography (CT) Scanner. BMC Med. Inform. Decis. Mak..

[14] Hummel M.J.M., Volz F., Van Manen J.G., Danner M., Dintsios C.M., Ijzerman M.J., Gerber A. (2012). Using the Analytic Hierarchy Process to Elicit Patient Preferences: Prioritizing Multiple Outcome Measures of Antidepressant Drug Treatment. Patient.

[15] Kwak N.K., McCarthy K.J., Parker G.E. (1997). A Human Resource Planning Model for Hospital/Medical Technologists: An Analytic Hierarchy Process Approach. J. Med. Syst..

[16] Ashour O.M., Okudan Kremer G.E. (2013). A Simulation Analysis of the Impact of FAHP-MAUT Triage Algorithm on the Emergency Department Performance Measures. Expert Syst. Appl..

[17] Abdel-Basset M., Manogaran G., Gamal A., Smarandache F. (2019). A Group Decision Making Framework Based on Neutrosophic TOPSIS Approach for Smart Medical Device Selection. J. Med. Syst..

[18] Malekpoor H., Mishra N., Sumalya S., Kumari S. (2017). An Efficient Approach to Radiotherapy Dose Planning Problem: A TOPSIS Case-Based Reasoning Approach. Int. J. Syst. Sci. Oper. Logist..

[19] Li D.P., He J.Q., Cheng P.F., Wang J.Q., Zhang H.Y. (2018). A Novel Selection Model of Surgical Treatments for Early Gastric Cancer Patients Based on Heterogeneous Multicriteria Group Decision-Making. Symmetry.

[20] Chung S., Kim S., Kim J., Sohn K. (2010). Use of Multiattribute Utility Theory for Formulary Management in a Health System. Am. J. Health Syst. Pharm..

[21] Abbas A., Bilal K., Zhang L., Khan S.U. (2015). A Cloud Based Health Insurance Plan Recommendation System: A User Centered Approach. Future Gener. Comput. Syst..

[22] Amaral T.M., Costa A.P.C. (2014). Improving Decision-Making and Management of Hospital Resources: An Application of the PROMETHEE II Method in an Emergency Department. Oper. Res. Health Care.

[23] Uzun D., Uzun B., Sani M., Helwan A., Nwekwo C., Veysel F., Sentürka N., Ozsahin I. (2017). Evaluating Cancer Treatment Alternatives Using Fuzzy PROMETHEE Method. Int. J. Adv. Comput. Sci. Appl..

[24] Maisaini M., Uzun B., Ozsahin I., Uzun D. (2019). Evaluating Lung Cancer Treatment Techniques Using Fuzzy Promethee Approach. Proceedings of the Advances in Intelligent Systems and Computing.

[25] Sarigül F., Hülagü S., Uzun Ozsahin D. (2021). Evaluation of Oral Antiviral Treatments for Chronic Hepatitis B Using Fuzzy PROMETHEE. Applications of Multi-Criteria Decision-Making Theories in Healthcare and Biomedical Engineering.

[26] Zeng Q.L., Li D.D., Yang Y. (2013). Bin VIKOR Method with Enhanced Accuracy for Multiple Criteria Decision Making in Healthcare Management. J. Med. Syst..

[27] Manupati V.K., Ramkumar M., Baba V., Agarwal A. (2021). Selection of the Best Healthcare Waste Disposal Techniques during and Post COVID-19 Pandemic Era. J. Clean. Prod..

[28] Chang T.H. (2014). Fuzzy VIKOR Method: A Case Study of the Hospital Service Evaluation in Taiwan. Inf. Sci..

[29] Bastani H., Bastani O., Sinchaisri W.P. (2021). Improving Human Decision-Making with Machine Learning. arXiv.

[30] Hsu J.-C., Wu F.-H., Lin H.-H., Lee D.-J., Chen Y.-F., Lin C.-S., Hsu J.-C., Wu F.-H., Lin H.-H., Lee D.-J. (2022). AI Models for Predicting Readmission of Pneumonia Patients within 30 Days after Discharge. Electronics.

[31] Arnaud E., Elbattah M., Gignon M., Dequen G. Deep Learning to Predict Hospitalization at Triage: Integration of Structured Data and Unstructured Text. Proceedings of the 2020 IEEE International Conference on Big Data.

[32] Casal-Guisande M., Comesaña-Campos A., Dutra I., Cerqueiro-Pequeño J., Bouza-Rodríguez J.-B. (2022). Design and Development of an Intelligent Clinical Decision Support System Applied to the Evaluation of Breast Cancer Risk. J. Pers. Med..

[33] Casal-Guisande M., Comesaña-Campos A., Cerqueiro-Pequeño J., Bouza-Rodríguez J.-B. (2020). Design and Development of a Methodology Based on Expert Systems, Applied to the Treatment of Pressure Ulcers. Diagnostics.

[34] Comesaña-Campos A., Casal-Guisande M., Cerqueiro-Pequeño J., Bouza-Rodríguez J.B. (2020). A Methodology Based on Expert Systems for the Early Detection and Prevention of Hypoxemic Clinical Cases. Int. J. Environ. Res. Public Health.

[35] Casal-Guisande M., Comesaña-Campos A., Pereira A., Bouza-Rodríguez J.-B., Cerqueiro-Pequeño J. (2022). A Decision-Making Methodology Based on Expert Systems Applied to Machining Tools Condition Monitoring. Mathematics.

[36] Kochenderfer M.J. (2015). Decision Making Under Uncertainty: Theory and Application.

[37] Celikyilmaz A., Türksen I.B. (2009). Modeling Uncertainty with Fuzzy Logic.

[38] Zadeh L.A. (1965). Fuzzy Sets. Inf. Control..

[39] Bellman R.E., Zadeh L.A. (1970). Decision-Making in a Fuzzy Environment. Manag. Sci..

[40] Gau W.L., Buehrer D.J. (1993). Vague Sets. IEEE Trans. Syst. Man Cybern..

[41] Atanassov K.T. (1999). Intuitionistic Fuzzy Sets.

[42] Bustince H., Burillo P. (1996). Vague Sets Are Intuitionistic Fuzzy Sets. Fuzzy Sets Syst..

[43] Xu Z., Yager R.R. (2006). Some Geometric Aggregation Operators Based on Intuitionistic Fuzzy Sets. Int. J. Gen. Syst..

[44] Xu Z. (2005). An Overview of Methods for Determining OWA Weights. Int. J. Intell. Syst..

[45] Thunnissen D.P. (2005). Propagating and Mitigating Uncertainty in the Design of Complex Multidisciplinary Systems. Ph.D. Thesis.

